# Supplementary material for: Developmental, regenerative, and behavioral dynamics in acoel reproduction
Source: eLife. 2026 Mar 20;14:RP105712. doi: 10.7554/eLife.105712 (PMC13004597; doi:10.7554/eLife.105712)
Supplement: MDAR checklist [file elife-105712-mdarchecklist1.pdf]

eLife

Westbrook Centre
Milton Road
Cambridge CB4 1YG, UK

P 01223 855340
W elifesciences.org
T @elife

Materials Design Analysis Reporting (MDAR) Checklist for Authors

The MDAR framework establishes a minimum set of requirements in transparent reporting mainly applicable to studies in the life sciences.

eLife asks authors to **provide detailed information within their article** to facilitate the interpretation and replication of their work. Authors can also upload supporting materials to comply with relevant reporting guidelines for health-related research (see EQUATOR Network), life science research (see the BioSharing Information Resource), or animal research (see the ARRIVE Guidelines and the STRANGE Framework; for details, see *eLife*'s Journal Policies). Where applicable, authors should refer to any relevant reporting standards materials in this form.

For all that apply, please note **where in the article** the information is provided. Please note that we also collect information about data availability and ethics in the submission form.

Materials:

Newly created materials	Indicate where provided: section/figure legend	N/A
N/A		N/A

Antibodies	Indicate where provided: section/figure legend	N/A
Tropomyosin, Piwi-1, FMRFamide, goat anti-rabbit secondary antibody	Methods	N/A

DNA and RNA sequences	Indicate where provided: section/figure legend	N/A
N/A		N/A

Cell materials	Indicate where provided: section/figure legend	N/A
N/A		N/A
		N/A

Experimental animals	Indicate where provided: section/figure legend	N/A
Hofstenia miamia, cultured in the lab.	Methods	
		N/A

Plants and microbes	Indicate where provided: section/figure legend	N/A
N/A		N/A
N/A		N/A

Human research participants	Indicate where provided: section/figure legend) or state if these demographics were not collected	N/A
N/A		N/A

Design:

Study protocol	Indicate where provided: section/figure legend	N/A
N/A		N/A

Laboratory protocol	Indicate where provided: section/figure legend	N/A
N/A		N/A

Experimental study design (statistics details) *		
**For in vivo studies: State whether and how the following have been done**	**Indicate where provided: section/figure legend. If it could have been done, but was not, write “not done”**	**N/A**
Sample size determination	**Selected based on feasibility and expectation of power**	
Randomisation	**Where appropriate - see Methods**	
Blinding	**N/A**	
Inclusion/exclusion criteria	**See Methods and code**	

Sample definition and in-laboratory replication	Indicate where provided: section/figure legend	N/A
N/A	See Methods	

**Ethics**	**Indicate where provided: section/submission form**	**N/A**
**N/A**		N/A
Studies involving experimental animals: State details of authority granting ethics approval (IRB or equivalent committee(s), provide reference number for approval.	**N/A**	
**N/A**		N/A

**Dual Use Research of Concern (DURC)**	**Indicate where provided: section/submission form**	**N/A**
**N/A**		N/A

Analysis:

**Attrition**	**Indicate where provided: section/figure legend**	**N/A**
**Exclusion criteria explained in text where appropriate**	**See Methods**	

**Statistics**	**Indicate where provided: section/figure legend**	**N/A**
Describe statistical tests used and justify choice of tests.	**See Methods.**	

**Data availability**	**Indicate where provided: section/submission form**	**N/A**
For newly created and reused datasets, the manuscript includes a data availability statement that provides details for access (or notes restrictions on access).	**All data and code available on Zenodo**	
When newly created datasets are publicly available, provide accession number in repository OR DOI and licensing details where available.		N/A
	**https://zenodo.org/records/16923213/**	

**Code availability**	**Indicate where provided: section/figure legend**	**N/A**
**See Data Availability statement and Zenodo repository**	https://zenodo.org/records/16923213/	
		N/A

		N/A

Reporting:

The MDAR framework recommends adoption of discipline-specific guidelines, established and endorsed through community initiatives.

Adherence to community standards	Indicate where provided: section/figure legend	N/A
N/A		N/A

---

* We provide the following guidance regarding transparent reporting and statistics; we also refer authors to [Ten common statistical mistakes to watch out for when writing or reviewing a manuscript].

Sample-size estimation

- You should state whether an appropriate sample size was computed when the study was being designed
- You should state the statistical method of sample size computation and any required assumptions
- If no explicit power analysis was used, you should describe how you decided what sample (replicate) size (number) to use

Replicates

- You should report how often each experiment was performed
- You should include a definition of biological versus technical replication
- The data obtained should be provided and sufficient information should be provided to indicate the number of independent biological and/or technical replicates
- If you encountered any outliers, you should describe how these were handled
- Criteria for exclusion/inclusion of data should be clearly stated
- High-throughput sequence data should be uploaded before submission, with a private link for reviewers provided (these are available from both GEO and ArrayExpress)

Statistical reporting

- Statistical analysis methods should be described and justified
- Raw data should be presented in figures whenever informative to do so (typically when N per group is less than 10)
- For each experiment, you should identify the statistical tests used, exact values of N, definitions of center, methods of multiple test correction, and dispersion and precision measures (e.g., mean, median, SD, SEM, confidence intervals; and, for the major substantive results, a measure of effect size (e.g., Pearson's r, Cohen's d)
- Report exact p-values wherever possible alongside the summary statistics and 95% confidence intervals. These should be reported for all key questions and not only when the p-value is less than 0.05.

Group allocation

- Indicate how samples were allocated into experimental groups (in the case of clinical studies,

please specify allocation to treatment method); if randomization was used, please also state if restricted randomization was applied

- Indicate if masking was used during group allocation, data collection and/or data analysis
